# Managing Dual Intracranial Pathologies of a Ruptured ACA Aneurysm Presenting Concomitantly with a Large Anterior Fossa Meningioma: Illustrative Case with Operative Video and Literature Review

**DOI:** 10.1055/a-2945-7117

**Published:** 2026-09-10

**Authors:** Aathmika Nandan, Justin Z. Wang, Ivan Radovanovic, Farshad Nassiri

**Affiliations:** 1 7938 Temerty Faculty of Medicine, The University of TorontoTorontoOntarioCanada; 2 Division of NeurosurgeryDepartment of Surgery 7938 University of TorontoTorontoOntarioCanada; 3 7989 MacFeeters Hamilton Neuro-Oncology Program, Princess Margaret Cancer Centre, University Health NetworkM5G 2C4TorontoOntarioCanada

**Keywords:** meningioma, aneurysm, ruptured, subarachnoid hemorrhage, planum sphenoidale

## Abstract

**Background** Meningiomas and intracranial aneurysms rarely co-occur and even less frequently present with acute subarachnoid hemorrhage (SAH) from aneurysm rupture. When anatomically adjacent, management requires nuanced decision-making.

**Observations** We present an illustrative case and video of a ruptured distal anterior cerebral artery dissecting aneurysm causing SAH and intraparenchymal hemorrhage in a patient with an anterior fossa meningioma encasing bilateral optic nerves. The aneurysm location, morphology, and potential involvement of an eloquent parent vessel branch deemed endovascular embolization and parent vessel sacrifice unsafe. The meningioma directly contacted the aneurysm and its parent artery, rendering pre-resection securement unfeasible. We performed a single-setting, open, frontotemporal craniotomy with early Sylvian fissure dissection for proximal control, followed by systematic tumor resection and microsurgical clipping of the A2 dissection. Gross total resection of the meningioma with clip ligation of the aneurysm and preservation of parent vessel/associated perforators was achieved. The patient returned to work 6 months postoperatively without persistent neurologic deficits. A narrative review of the literature on aneurysmal SAH associated with concomitant meningioma was conducted.

**Lessons** This case highlights the intricacies of managing vascular and neuro-oncologic pathology presenting simultaneously in a patient in extremis, along with a literature review. The patient consented to the procedure and publication.

## Introduction

Meningiomas and intracranial aneurysms are independently common neurosurgical pathologies, yet their co-occurrence is uncommon, and their simultaneous presentation with an acute intracranial hemorrhage is rarer still. Individually, each pathology carries well-established management principles; however, for a patient in extremis where both tumor and aneurysm are anatomically adjacent to one another, a time-sensitive clinical challenge arises in determining (1) how each pathology should be treated, (2) what is the optimal timing of treatment, (3) which pathology should be addressed first, and (4) what preoperative or intraoperative technical considerations should be made to maximize safety. Here, we present an illustrative case, including operative video discussing each of these points, as well as a literature review on previously reported cases of aneurysmal rupture in patients with concomitant meningioma.

## Illustrative Case

A 52-year-old male presented with sudden-onset headache, left-arm weakness, nausea, and vomiting. He was drowsy and confused in the emergency department but was still able to follow commands. A formal visual acuity and visual field exam was unable to be performed reliably due to the patient’s neurological status; however, the patient was able to count fingers in both eyes and did not appear to have any obvious visual field deficits to confrontation.

### Investigation

**Fig. 1:**
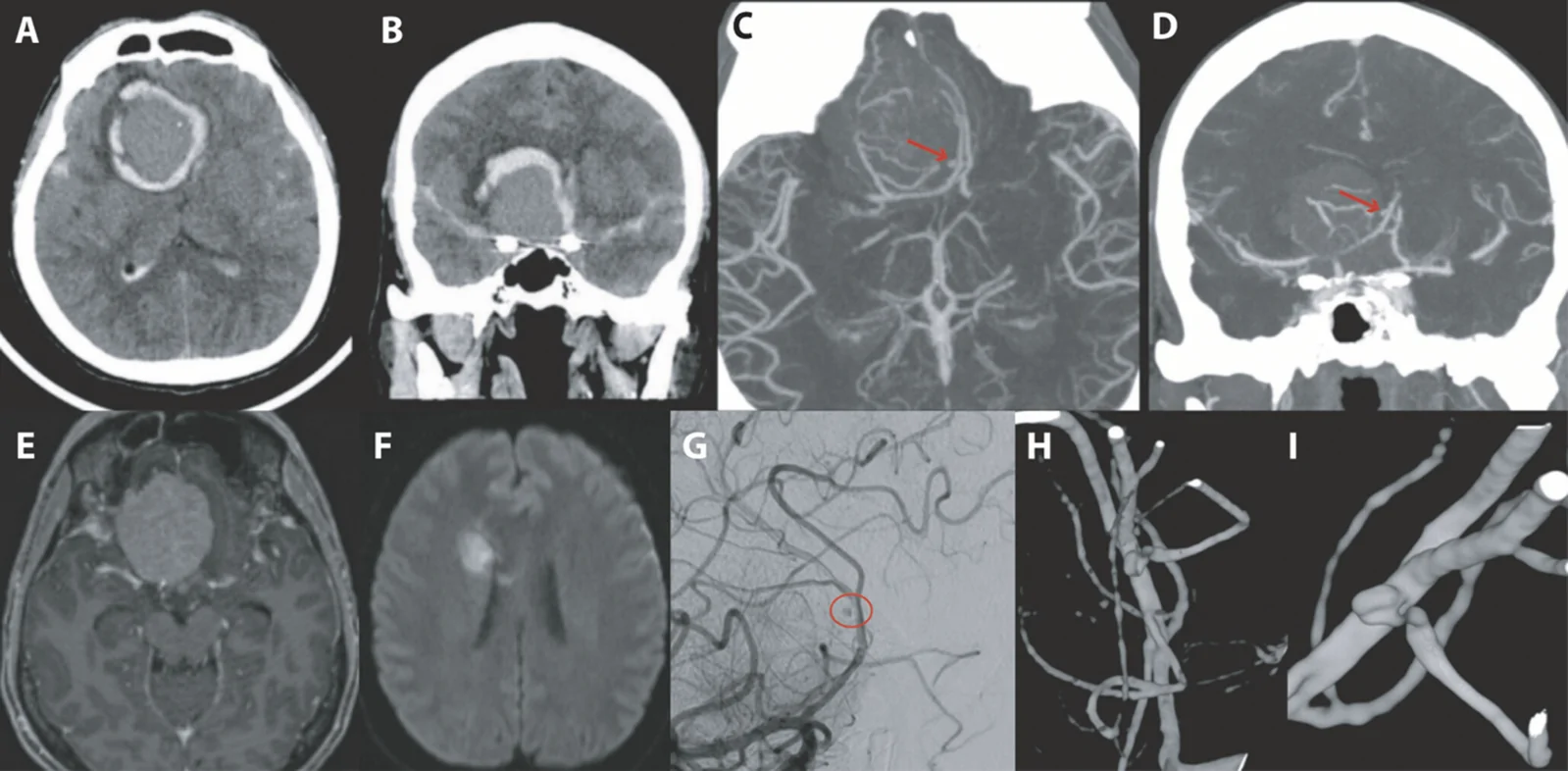
Preoperative imaging. ( **A, B** ) Non-contrast CT demonstrating intraparenchymal hemorrhage around a large anterior skull base mass and basal subarachnoid hemorrhage extending into the Sylvian fissures bilaterally and the interhemispheric fissure. ( **C, D** ). Maximal intensity projections on CTA demonstrating displacement of the bilateral ACAs by the meningioma and the associated aneurysm from the right A2 (red arrow) on the medial margin of the tumor. ( **E** ) MRI with gadolinium demonstrating the large anterior fossa homogenously enhancing lesion suggestive of meningioma. ( **F** ) Diffusion weighted imaging demonstrating a preoperative right caudate infarct. ( **G** ) Digital subtraction angiogram (DSA) lateral projection of a right ICA injection demonstrating the saccular aneurysm arising from the right A2 (red circle). ( **H, I** ) Three-dimensional reconstructions from the DSA redemonstrating the irregular saccular aneurysm/pseudoaneurysm arising from a dysplastic origin of the frontopolar branch of the ACA. ACA, anterior cerebral artery; CT, computed tomography; CTA, CT angiography; ICA, internal carotid artery; MRI, magnetic resonance imaging.

An urgent non-contrast computed tomography (CT) scan of the head revealed a large extra-axial mass centered on the planum sphenoidale, slightly right to the midline. Significant vasogenic edema was noted, along with acute hemorrhage along the periphery of the mass and thick basal subarachnoid hemorrhage (SAH) ( Fig. 1A, B ). CT angiogram demonstrated a 2-mm saccular aneurysm from the right A2 segment of the anterior cerebral artery (ACA) with an irregular dome configuration abutting the medial margin of the mass ( Fig. 1C, D ).

Magnetic resonance imaging (MRI) of the brain showed a large extra-axial mass arising from the anterior cranial fossa encasing the right optic nerve and compressing the left optic nerve ( Fig. 1E ). An acute right caudate infarct was seen on diffusion weighted imaging ( Fig. 1F ). MR time-of-flight angiography re-demonstrated this likely meningioma causing displacement of the A2 segments bilaterally and contacting the above-described aneurysm.

We reviewed the case with our Interventional Neuroradiology colleagues, and a digital subtraction angiogram (DSA) was obtained urgently to determine whether the aneurysm was amenable to endovascular treatment ( Fig. 1G–I ). The DSA and three-dimensional reconstruction allowed for improved visualization of the neck and origin of the aneurysm. This revealed that the likely dissecting aneurysm arose from a dysplastic origin of a frontopolar branch of the ACA, which was partially feeding both the tumor and frontal parenchyma ( Fig. 1G–I ).

### Rationale for Microsurgical Management

After detailed discussion of the case with our Interventional Neuroradiology team, several features of the aneurysm made it high risk for endovascular treatment, including its small size, distal location, and its possible pseudoaneurysm nature without a true wall, which would require vessel sacrifice of a potentially eloquent branch with embolization or coiling. Therefore, open microsurgical clipping was advised and selected as our safest approach. Given the patient’s drowsiness, substitute decision-maker informed consent was obtained from the patient’s spouse to proceed with the urgent operation. Risks of surgery disclosed included death, intraoperative and post-operative re-bleeding, parenchymal injury, ACA injury and infarct, clip malplacement requiring re-positioning, unilateral or bilateral anosmia, hydrocephalus, cerebrospinal fluid (CSF) leak, wound infection, and post-operative seizures.

### Alternatives Considered and Rationale for Their Exclusion

1. **Endovascular coiling of the aneurysm prior to craniotomy** : It was not favored due to reasons described above, which included high risk of intraprocedural rupture and the need to occlude or embolize either the parent artery or orbitofrontal branch of the ACA to secure the aneurysm.
2. **Open microsurgical clipping before tumor resection** : It was not favored due to the requirement for significant frontal lobe retraction and increased mass effect on the bilateral optic nerves, which were already compressed by the tumor to reach the aneurysm. Additionally, the tumor was directly compressing and obscuring view of the aneurysm neck, orbitofrontal branch, and contralateral A2, precluding safe clipping. A two-staged operation with a contralateral (left) craniotomy for aneurysm clipping followed by an ipsilateral (right) craniotomy for meningioma resection in potentially a delayed fashion was considered as well but not selected given the potential difficulties in dissecting the tumor away from the A1s bilaterally for proximal control and to preserve the adjacent potential en passage parent vessel to eloquent brain that may have been involved with the tumor as well. Furthermore, this would have exposed the patient to the risks of two craniotomies and potential injury to bilateral hemispheres.
3. **Tumor resection with aneurysm securement as a second stage** : It was not favored given the persistent risk of re-rupture of an already ruptured aneurysm if not secured and the potential need for hypertensive treatment of vasospasm later on.
4. **Aneurysm clipping without tumor resection** : It was not favored given the above features of accessing the aneurysm surgically, and the fact that leaving the tumor would contribute to the patient’s already symptomatic, elevated intracranial pressure (ICP), and deferring an operation that the patient would ultimately need in the future when access has already been achieved.

### Operative Sequence



**Video 1** Meningioma A2 aneurysm. A2, anterior cerebral artery A2 segment; CTA, CTA, computed tomography angiography; DSA, digital subtraction angiogram; MRI, magnetic resonance imaging.


Given that the meningioma directly compressed and obscured the neck of the aneurysm, securement of the aneurysm before tumor resection, although preferred given that it would mitigate the risks of intraoperative re-rupture, was not possible without at least some degree of tumor debulking, particularly given the tumor’s already significant mass effect on the brain and optic apparatus, and the patient’s young age in the face of significantly elevated intracranial pressure due to his subarachnoid hemorrhage. Attempting to reach the aneurysm prior to tumor debulking would likely require an unsafe degree of frontal retraction and retraction on the optic nerves. Therefore, we planned to obtain proximal control at the level of the internal carotid artery (ICA) and ipsilateral A1 prior to initiating tumor resection. Our operative plan and intraoperative video are presented in further detail in Video 1 . The primary goals of surgery included (1) securement of the ruptured aneurysm to prevent re-rupture while preventing injury to the parent vessel and associated perforators, and (2) maximal safe resection of the intracranial meningioma to facilitate the former.

**Fig. 2:**
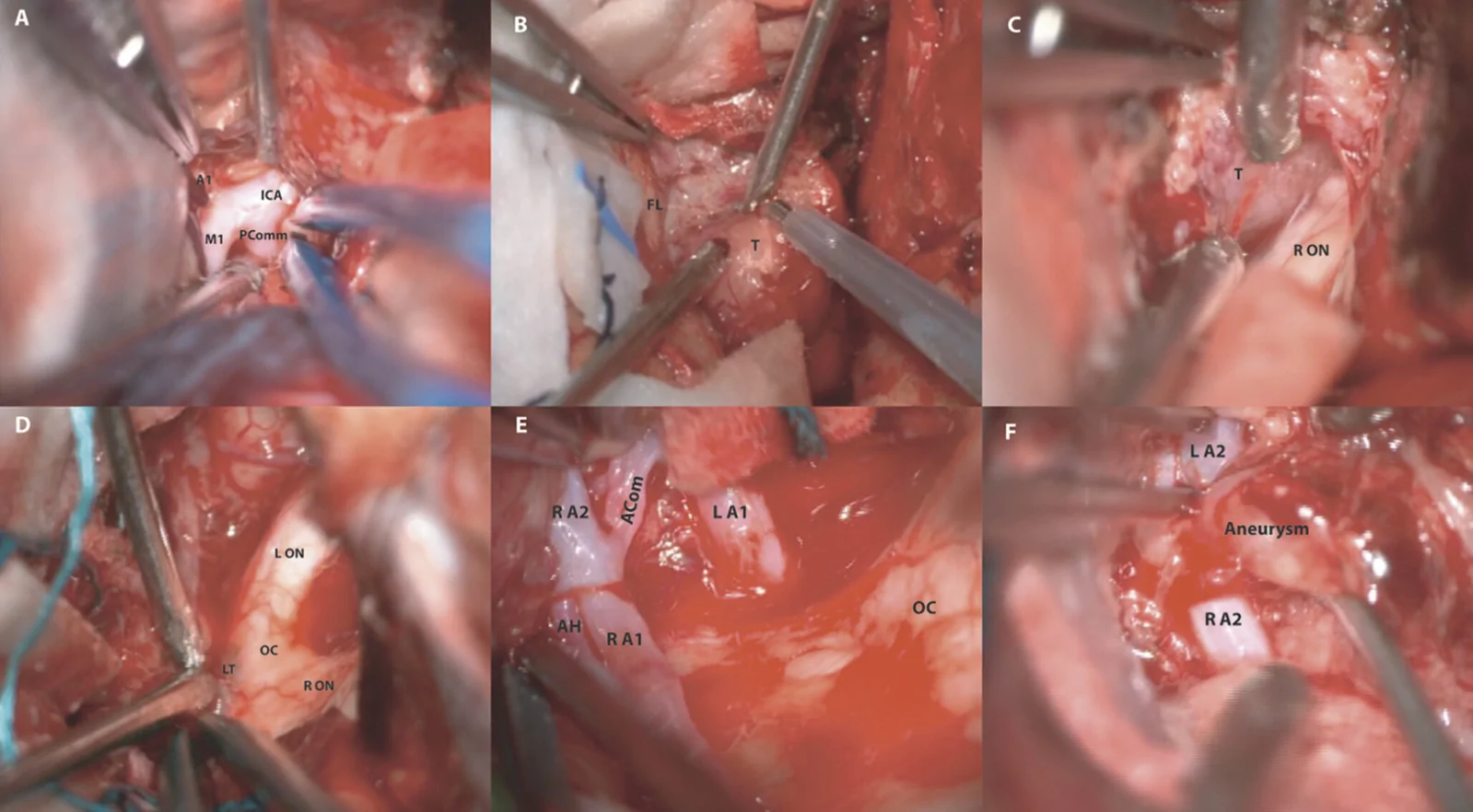
Intraoperative imaging of key surgical steps (see Video 1 ). ( **A** ) Following proximal Sylvian fissure dissection to gain proximal control prior to tumor resection. ( **B** ) Tumor debulking using the ultrasonic aspirator and extracapsular dissection from the frontal lobe. ( **C** ) Dissection of tumor from the ipsilateral optic nerve. ( **D** ) View following tumor resection of the bilateral optic nerves, optic chiasm, and laminal terminalis, which is subsequently opened. ( **E** ) Interhemispheric dissection following the proximal bilateral ACAs to the AComm and A2 to locate the aneurysm. ( **F** ) Identification of the aneurysm arising from the ipsilateral A2 segment and dissection of the neck. A1, anterior cerebral artery A1 segment (R, right; L, left); A2, anterior cerebral artery A2 segment; ACA, anterior cerebral artery; AComm, anterior communicating artery; AH, recurrent artery of Heubner; FL, frontal lobe; ICA, internal carotid artery; LT, lamina terminalis; M1, middle cerebral artery M1 segment; OC, optic chiasm; ON, optic nerve (R, right; L, left); PComm, posterior communicating artery; T, tumor.

Operative adjuncts utilized included the operative microscope, stereotactic frameless navigation for tumor resection and for potential ventriculostomy, neuromonitoring including somatosensory-evoked potentials and motor-evoked potentials for temporary clipping, indocyanine green for intraoperative angiography, and intraoperative Doppler to confirm the patency of parent vessels. Key operative steps as detailed and followed ( Video 1 ). First, a standard pterional craniotomy was performed, with the patient in supine position, head turned 30 degrees to the contralateral side and extended with the malar eminence at the highest point and pinned. Next, extradural drilling of the sphenoid ridge, orbital roof, and middle fossa floor was performed with a 5-mm cutting burr. Once the dura was open, a proximal Sylvian fissure split was performed with identification and control of the ICA ( Fig. 2A ). The ICA was followed to its bifurcation and ipsilateral A1 to establish proximal control. Next, the tumor was dissected from the frontal lobe, identifying an area on its capsule to open, followed by generous intracapsular tumor debulking using the ultrasonic aspirator ( Fig. 2B ). Progressive debulking enabled extracapsular dissection of the tumor, including microsurgical dissection of the tumor off the optic apparatus bilaterally and off its origin along the anterior fossa, where some areas of focal hyperostosis required drilling with a 3-mm diamond ( Fig. 2C ). Following complete tumor resection, the lamina terminalis was opened to enable further brain relaxation to facilitate opening of the interhemispheric fissure ( Fig. 2D, E ). The ipsilateral A1 was followed to the anterior communicating artery (AComm), then the contralateral A1, ipsilateral A2, and the interhemispheric fissure was opened to identify the contralateral A2 in that order ( Fig. 2E ). The ipsilateral A2 was followed more distally to identify the dysplastic aneurysm ( Fig. 2F ). The neck of the aneurysm was dissected out, including identifying the origin of the orbitofrontal artery at its origin and the contralateral A2 to facilitate safe microsurgical clipping. Postclipping, intraoperative ICG and Doppler were utilized to confirm complete occlusion of the aneurysm and patency of the parent arteries and their branches. The dura of the anterior fossa was coagulated with the bipolar cautery to achieve a Simpson grade 2 resection prior to closure.

Importantly, if intraoperative rupture were to have occurred at any point during tumor resection, resection would have been paused, a temporary clip would have immediately been placed on the ipsilateral A1, which would have been followed to the AComm, and contralateral A1 (another temporary clip would be placed here if bleeding continued), and dissection and securement of the aneurysm would have proceeded as described above. If needed for exposure, resection of the gyrus rectus would have been undertaken to reach the aneurysm to avoid significant retraction on the frontal lobes and downward traction on the optic nerves if a significant amount of tumor remained.

### Outcomes

**Fig. 3:**
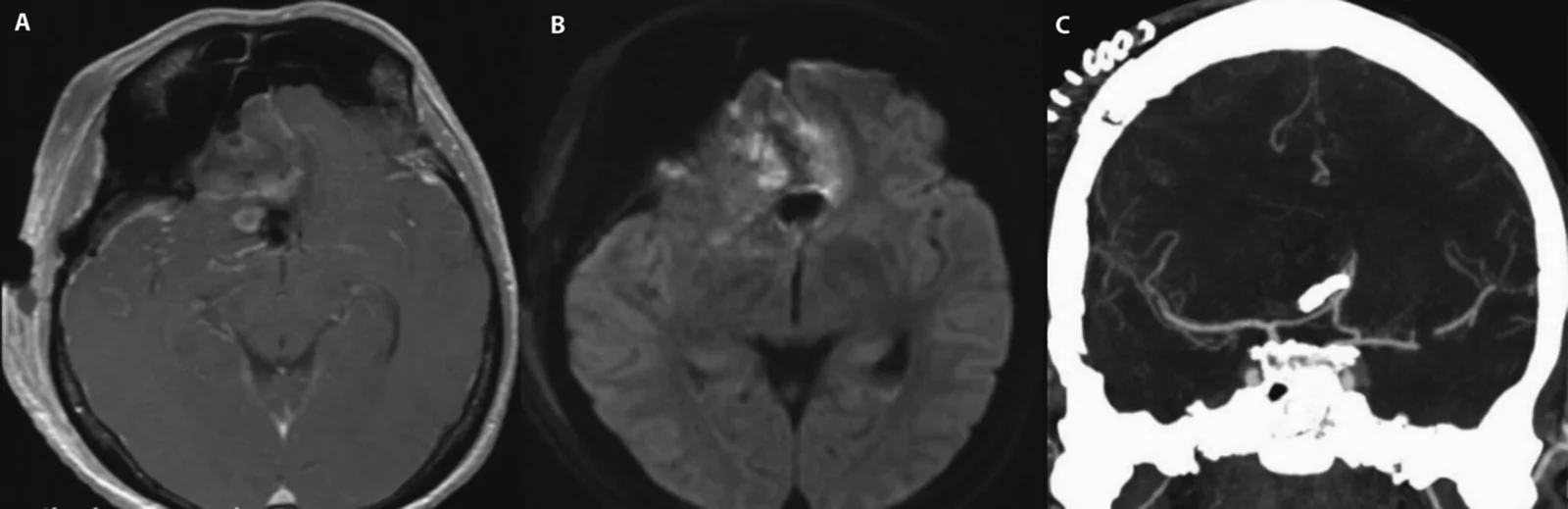
Postoperative imaging. ( **A** ) MRI T1W post-gadolinium demonstrating gross total resection of the meningioma without any obvious residual tumor. ( **B** ) Diffusion weighted imaging demonstrating no new infarcts postresection and aneurysm clipping. ( **C** ) CTA demonstrating complete clip ligation of the aneurysm with patency of bilateral A2s distally. A1, anterior cerebral artery A1 segment (R, right; L, left); A2, anterior cerebral artery A2 segment; CTA, computed tomography angiography; MRI, magnetic resonance imaging.

Gross total resection of the meningioma was achieved along with successful clip ligation of the aneurysm, with preservation of the parent vessel and the orbitofrontal branch ( Fig. 3A–C ). The patient was successfully extubated after surgery and remained in hospital for 14 days to monitor for vasospasm. His left-sided weakness, present preoperatively, improved during his hospital stay. Although he developed radiographic vasospasm, clinically he remained stable and was discharged home with outpatient rehabilitation. At his 6-month follow-up, the patient returned to work in his manual construction job.

## Discussion

### Observations

The central observation in this case was the rare co-occurrence of a meningioma and a ruptured intracranial aneurysm, presenting with an acute subarachnoid and intraparenchymal hemorrhage. Both pathologies were anatomically adjacent to one another, and the aneurysm arose from the dysplastic origin of a branch of the ACA supplying eloquent frontal parenchyma, rendering standard endovascular treatment risky. An operative sequence of proximal vascular control, tumor resection, and finally microsurgical clipping was chosen as the safest option to address both pathologies in a time-sensitive manner for this patient.

The prevalence of intracranial aneurysms in patients with brain tumors has been proposed to range from 0.7% to 7.7% 1 ; however, the true rate may be underestimated as preoperative vascular imaging is not routinely performed in many cases. 1 The management of meningiomas with concomitant aneurysms remains a challenge, with no consensus on the best approach, which is largely case dependent based on patient presentation and the location of the two pathologies in association with one another. 2 3 Incidental aneurysms remote from a presenting meningioma may be followed clinically and radiographically without treatment or electively treated if they meet a size threshold or demonstrate interval growth on imaging. Similarly, incidental meningiomas in patients with aneurysms to be treated can usually be dealt with in a similar manner. However, the management paradigm changes dramatically when patients present with hemorrhage from their ruptured aneurysm and the associated aneurysm is anatomically associated with the meningioma in question, requiring urgent decision-making to address both pathologies sequentially or simultaneously within a narrow window of time, as presented in this case.

The primary question is usually whether to secure the aneurysm first or resect the tumor. In a retrospective study, Fischer et al. found that tumor resection was prioritized in 58 cases and aneurysm securement was prioritized in 38 cases. 4 This reflects the absence of consensus or standardized protocol for managing the co-occurrence of these pathologies and truly case-by-case decision-making.

### Unruptured Aneurysm Concomitant with Meningioma

Unruptured intracranial aneurysms that coexist with meningiomas can be managed in accordance with standard guidelines for unruptured intracranial aneurysms, as the existence of a meningioma generally does not change the rupture risk of a spatially unrelated aneurysm. 4 5 It has been proposed that increased directional blood flow to meet blood supply demands of highly vascular meningiomas can result in abnormal hemodynamic stress on tumor-feeding arteries and induce secondary changes in the arterial wall that may contribute to the formation of aneurysms. 6

Conservative aneurysm management appears generally safe for small aneurysms that are contralateral to the tumor, or when ipsilateral aneurysms do not interfere with the tumor resection. However, initial treatment of the aneurysm surgically or endovascularly prior to meningioma resection is preferred for ipsilateral, symptomatic, or large aneurysms. 1 Furthermore, the same aneurysm features associated with rupture risk independently are also associated with increased risk of intraoperative rupture during tumor resection, including those larger than 7 mm, the presence of a daughter sac or bleb, AComm or posterior communicating artery (PComm) location, 7 and, expectedly, if tumor resection is expected to cause direct manipulation of the vessel that contains the aneurysm. These features generally mandate treatment of the aneurysm first prior to tumor resection. In particular, presence of an aneurysm in close proximity to or encased within a meningioma makes the excision of the tumor extremely hazardous without first securing the aneurysm. 8

When the two lesions are near one another anatomically, a single surgery can effectively address both pathologies simultaneously. However, a two-stage approach with preoperative endovascular embolization/coiling followed by a craniotomy is also viable and is generally favored for aneurysms that arise from feeder arteries. 3

### Ruptured Aneurysm Concomitant with Meningioma

Table 1 presents case reports of ruptured aneurysms concomitant with meningiomas. Seventeen cases dealt with the tumor and the aneurysm in a single open surgery; this included 13 cases of single-stage clipping and gross total resection, 1 case of gross total resection and coagulation of aneurysm, 1 case of gross total resection and surgical ICA trapping and bypass, and 2 en bloc resections. Three cases took a two-stage approach; one case underwent preoperative endovascular embolization followed by gross total resection, and two cases had a resection of the tumor followed by endovascular coil embolization of the aneurysm. One case took a three-stage approach of gross total resection followed by stereotactic radiotherapy, followed by coil embolization of the aneurysm. Three cases did not receive treatment. When a ruptured aneurysm and meningioma present simultaneously, the symptomatic lesion should be prioritized. 5 Accordingly, intracranial aneurysms presenting with rupture and subarachnoid hemorrhage should be ideally treated before or simultaneously at the same time as tumor resection (as in our case here), given that the risk of rebleeding after an aneurysmal subarachnoid hemorrhage is associated with high morbidity and mortality. 30 However, establishing the source of the hemorrhage between aneurysmal subarachnoid hemorrhage and meningioma-related hemorrhage becomes an important step in diagnosis and management, given the sometimes highly vascular nature of some meningiomas. 3 Distinguishing between the two etiologies typically relies on the anatomical location of the hemorrhage and the aneurysm. In our case, the hemorrhage was attributed to the aneurysm given that the hemorrhage was centered around the aneurysm itself. The aneurysm arose from a branch point of an anatomically normal orbitofrontal artery (branch of A2), rather than from a specific feeder artery to the meningioma.

**Table 1:** Case reports of ruptured aneurysms concomitant with meningiomas, their respective locations, relationship to one another, and the intervention used.

Study	Meningioma location	Aneurysm location (number)	Meningioma–aneurysm relationship	Intervention
Algburi et al., 2022 9	Frontal convexity	Acomm (1)	Adjacent	Single stage: GTR + clipping
AlMutairi et al., 2026 10	Olfactory groove/planum sphenoidale	ACA (1)	Feeding artery	Two-stage: GTR + endovascular embolization of aneurysm
Alnaami et al., 2013 11	Falcine	ACA (1)	Embedded	Two-stage: preop endovascular embolization + GTR
Atallah et al., 2025 12	Planum sphenoidale	ICA (1)	Distant	Single stage: clipping + GTR
Balasubramanian et al., 2022 13	Frontal convexity, parasagittal	ACA (1)	Adjacent	Single stage: GTR + coagulation of aneurysm
Bloomgarden et al., 1987 14	Clinoid	ICA (1)	Adjacent	Single stage: clipping + GTR
De Bonis et al., 2023 15	Sphenoid wing	MCA (1)	Embedded-Feeding artery	Single stage: GTR
Feng et al., 2017 16	Tuberculum sella	ICA (2)	Adjacent	Single stage: clipping + GTR
Hoya et al., 2011 17	Clinoid	ICA (1)	Encased	Three-stage: GTR + SRT + coil embolization of aneurysm
Javalkar et al., 2009 18	Sphenoid wing	Acomm (1)	Distant	Single stage: clipping + GTR
Kandel et al., 1986 19	Frontal	MCA (1)	Embedded	Single stage: GTR
Licata et al., 1986 20	Frontal	Acomm (5)	Adjacent	No treatment
Licata et al., 1986 20	Infratentorial	Acomm (1)	Distant	No treatment
Licata et al., 1986 20	Frontal	Acomm (1)	Adjacent	Single stage: clipping + GTR
Licata et al., 1986 20	Infratentorial	Acomm (1)	Distant	No treatment
Meguins et al., 2017 21	Falcine	ACA (1)	Adjacent	Single stage: clipping + GTR
Najjar et al., 2007 22	Sphenoid wing	Acomm (1)	Adjacent	Single stage: clipping + GTR
Ogino et al., 1999 23	Tuberculum sellae	Acomm (1)	Embedded	Single stage: GTR + clipping
Okuyama et al., 2023 24	Parasellar	ICA (1)	Embedded	Single stage: GTR + surgical ICA trapping and bypass
Sharma et al., 2019 25	Clinoid	Acomm (1)	Adjacent	Single stage: GTR + clipping
Tanaka et al., 2022 26	Frontal convexity	ICA (1)	Feeding artery	Two-stage: GTR + endovascular coil embolization.
Waqas et al., 2015 27	Clinoid	ICA (1)	Adjacent	Single stage: GTR + clipping
Yang and Huang, 2014 28	Sphenoid wing	ICA (2)	Embedded	Single stage: GTR + clipping
Zhou et al., 2017 29	Clinoid	Pcomm (2)	Adjacent	Single stage: clipping + GTR

Abbreviations: ACA, anterior cerebral artery; AComm, anterior communicating artery; GTR, gross total resection; ICA, internal carotid artery; MCA, middle cerebral artery; SRT, stereotactic radiotherapy

If open surgery is selected, the anatomical relationship between the two lesions then determines the surgical approach. When the aneurysm and meningioma are near each other and can be reached through a single approach, a single-stage operation is expectedly preferred. For instance, in a systematic review by Abou-Mrad et al., 15 of 16 treated patients presenting with a ruptured aneurysm and meningioma underwent a single surgical procedure that addressed both pathologies. 3 Ideally, the aneurysm should be secured prior to tumor excision; however, when the meningioma directly obscures the neck of the aneurysm, a piecemeal decompression may be necessary to get adequate access. 8 In contrast, when the two lesions are contralateral and cannot be addressed simultaneously, the ruptured aneurysm should be dealt with first, with the meningioma deferred to a separate elective procedure given that the risk of aneurysm re-rupture far outweighs the risks of tumor progression. 31

### Lessons

Our case highlights an important clinical challenge of dual intracranial pathologies associated with one another in the face of a patient presenting in extremis. Although classic teaching and safe practices dictate that the ruptured aneurysm should be treated first, in our case, given the higher risks of endovascular treatment, the tumor’s direct compression and obscuration of the neck of the aneurysm, pre-resection aneurysm securement was not possible. Therefore, if tumor resection or debulking needs to be completed first, there are several key steps that must be adhered to maximize safety, including strict blood pressure control, early proximal control intraoperatively, and planning for contingency measures in case of intraoperative rupture during resection. We support our management with a literature review that reinforces the case-by-case decision-making that must be done in these instances depending on the idiosyncratic nature of the patient and pathology’s anatomy, their clinical status, and feasibility of endovascular treatment. We hope that our case and associated operative video will provide a reference for practitioners who may face this clinical scenario in their practice and can apply the same decision-making and intraoperative paradigms that we utilized to successfully manage these concomitant pathologies.
